# Application of autologous endometrial mesenchymal stromal/stem cells increases thin endometrium receptivity: a case report

**DOI:** 10.1186/s13256-020-02515-5

**Published:** 2020-10-17

**Authors:** I. M. Sapozhak, О. S. Gubar, A. E. Rodnichenko, A. V. Zlatska

**Affiliations:** 1 Biotechnology Laboratory Ilaya.Regeneration, Medical Company Ilaya, I. Kramskogo Street, 9, Kiev, 03115 Ukraine; 2 Biotechnology Laboratory, Medical Company “Good Cells”, I. Kramskogo Street 9, Kiev, 03115 Ukraine; 3 Institute of Molecular Biology and Genetics, National Academy of Sciences of Ukraine, Zabolotnogo Street, 150, Kiev, 03143 Ukraine; 4 State Institute of Genetic and Regenerative Medicine, National Academy of Medical Sciences of Ukraine, Vyshgorodskaja Street, 67, Kiev, 04114 Ukraine

**Keywords:** Endometrial receptivity, Endometrial mesenchymal stem/stromal cells, Thin endometrium

## Abstract

**Background:**

Pregnancy in cycles with the use of assisted reproductive technologies is possible only with the availability of good-quality embryos and a healthy receptive endometrium. The problem of lack of sensitivity of the endometrium is related to the syndrome of thin endometrium, which is caused by a number of factors. However, there is no single protocol for the treatment of this syndrome, the return/improvement of normal functionality of endometrial tissue, and obtaining the desired pregnancy.

**Case presentation:**

We report a case of a 38-year-old Ukrainian woman with a number of unsuccessful tries at pregnancy in cycles with the use of assisted reproductive technologies. We describe a clinical case of the use of mesenchymal stem cells of the human endometrium for a woman with thin endometrial syndrome to increase its receptivity for pregnancy. The basic steps of patient management, protocol of sampling material for obtaining a cell product based on endometrial stem cells, their basic morphofunctional characteristics, and post-treatment procedures to obtain the desired pregnancy are described.

**Conclusion:**

Application of autologous endometrial mesenchymal stem cells increases endometrial receptivity and the chance for pregnancy with use of assisted reproductive technologies.

## Introduction

A thin endometrium remains the most common uterine infertility factor. In most cases, a thin endometrium is defined as ≤ 7 mm by ultrasound examination. However, the definition of “thin” varies between ≤ 6–8 mm [7]. Its incidence increases with age and reaches 25% in women older than 40 years of age in natural cycles [4]. In *in vitro* fertilization (IVF) cycles, the percentage of thin endometrium is much lower, about 2.5%. However, these data are likely underestimated, because only cycles that proceed to embryo transfer are taken into account, whereas most IVF cycles with thin endometrium are to be canceled [6].

Different factors lead to decreased endometrial thickness. Traditionally, they are classified as inflammatory, iatrogenic, and idiopathic [7]. Despite the variability of causes, the outcomes of this state are similar: decreased implantation and pregnancy rates and increased miscarriage frequency.

The treatment of thin endometrium remains an ongoing challenge. Currently, there is no reliable, clinically approved strategy, although many approaches have been tried. These include hysteroscopic adhesiolysis, estrogen- and gonadotropin-releasing hormone agonist administration, vasoactive chemical measures (for example, aspirin, vitamin E, pentoxifylline, l-arginine, sildenafil), and intrauterine infusion of growth factor such as granulocyte-macrophage colony-stimulating factor, among others. In most cases, these measures have no or little effect [3, 5]. Currently, the use stem cell therapy seems to be the most promising approach, despite the lack of large-scale randomized clinical trials. We report a live birth case after the application of endometrial mesenchymal stem/stromal cells (MSCs) in a patient with a thin endometrium and a previous miscarriage history.

## Case presentation

Our patient was a 38-year-old Ukrainian woman who provided informed consent to report her clinical details and data of the case. The patient’s height was 171 cm, and her weight was 73 kg. Her menarche was at the age of 14. She had experienced menstrual irregularities for the last 5 years. In 2013, she had consulted a gynecologist about the absence of pregnancy and her menstrual irregularities. She was examined at that time and was diagnosed with primary infertility, endocrine; polycystic ovary syndrome; oligomenorrhea; insulin resistance; and moderate cervical dysplasia. The patient took oral contraceptives with antiandrogenic effect and a hypoglycemic agent from the biguanide group (metformin). Loop excision of the cervix was performed after preliminary treatment of the infection. Also, the patient underwent metrosalpingography, and her fallopian tubes were passable. In 2016, an ultrasound examination of the pelvic organs revealed an endometrial polyp. Hysteroscopy and polypectomy were performed. The diagnosis was histologically confirmed. In January 2017, the patient’s ovulation was stimulated with selective modulators of estrogen receptors, after which pregnancy occurred. At 10 weeks of pregnancy, an undeveloped pregnancy that corresponded to 6 weeks was detected by ultrasound. A manual vacuum was carried out to aspirate the conception product from the uterine cavity with subsequent administration of an antibiotic. Since 2017, the patient had taken progesterone and cyproterone acetate in the second phase of the menstrual cycle. Menstruation was scarce from 2017 and regular only as a result of the hormone drug use.

In 2018, the patient was referred to our clinic and complained of a lack of pregnancy for 1 year with a delay in menstruation of up to 2–3 months. Her menstruation was irregular, every 28–90 days for 3–4 days, painless, and scanty over the last year.

Examination with a speculum revealed that the cervical epithelium was not damaged; the discharge was milky in moderate quantities. Vaginal examination revealed the uterus in anteflexion; it was normal-sized, dense elastic, mobile, and painless. The patient’s ovaries were palpated on both sides, slightly enlarged, of limited mobility, and painless. Laboratory test results were as follows: antimullerian hormone 10.5 ng/ml (normal value 0.4–6.96), follicle-stimulating hormone 6.9 mIU/ml (normal value in the follicular phase 3.5–12.5), luteinizing hormone 11.8 mIU/ml (normal value in the follicular phase 1–11.4), estrogen 50.6 pg/ml (normal value in the follicular phase 13.6–190.4), prolactin 157 mIU/ml (normal value in the follicular phase 69–750), progesterone 0.18 ng/ml (normal value in the luteal phase 1.83–23.9), and insulin resistance index (homeostatic model assessment of insulin resistance) 3.5 (normal value < 3). The data obtained from a coagulogram, liver tests, glucose in blood serum, complete blood count, urinalysis, cervical cytology, and microscopy of a urogenital smear corresponded to the normal values. Human immunodeficiency virus, hepatitis B virus, hepatitis C virus, syphilis, and vertically transmitted infections were not detected. Pelvic ultrasound data on the 33rd day of the menstrual cycle were as follows: The uterus was in anteflexion, not displaced, not enlarged, and 5.0 cm in length; the anteroposterior diameter was 3.9 cm; the transverse diameter was 4.7 cm; and the volume was 4.8 cm^3^. The patient’s myometrium was homogeneous. Her endometrium was 0.4 cm and homogeneous. Her cervix length was 2.8 cm, width was 2.1 cm, and uniform. Her cervical canal was not visually changed. Her right ovary measured 4.6 × 2.2 × 3.8 cm with volume 14.6 cm^3^, enlarged; 12 follicles were located in the periphery with a maximum diameter of 0.5 cm. The patient’s left ovary measured 3.7 × 1.9 × 2.9 cm with a volume of 10.3 cm^3^, enlarged; 10 follicles were located in the periphery with a maximum diameter of 0.5 cm. The patient’s uterine tubes were not visualized. Free fluid in the retrouterine space was not detected. We concluded that the patient had ultrasound evidence of multifollicular ovaries. She was diagnosed with secondary infertility of endocrine genesis; hypothalamic-pituitary dysfunction, polycystic ovary syndrome, insulin resistance, and oligomenorrhea.

**Fig. 1:**
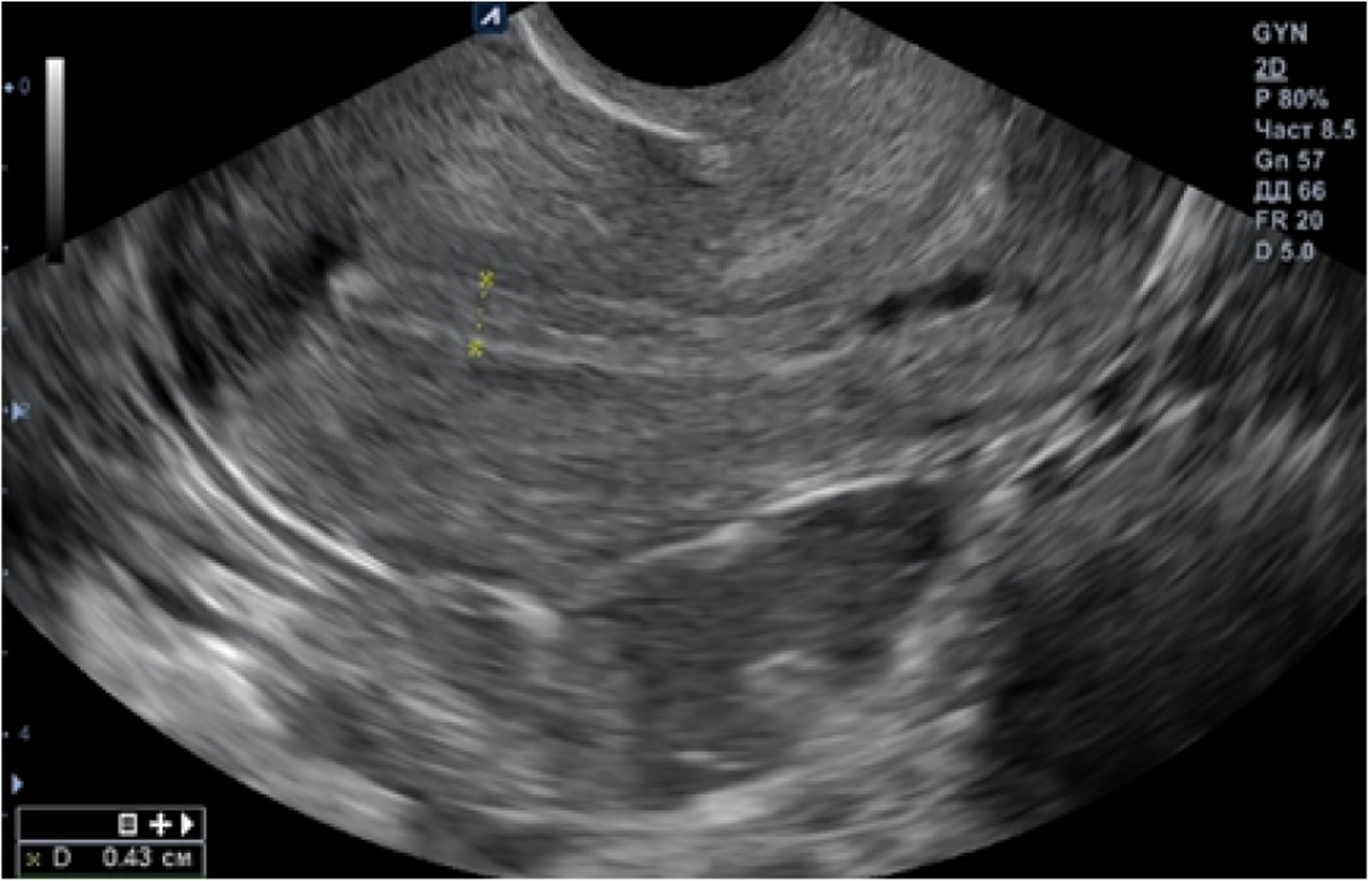
Ultrasound examination showing endometrial thickness during the preparation for embryo transfer

IVF was proposed. In August 2018, ovulation was stimulated with gonadotropins according to the short protocol, after which 12 embryos were obtained and cryopreserved on day 5, because according to ultrasound, the thickness of the endometrium was 0.4 cm. During subsequent consultations, the patient complained that her menstruation was scarce and lasted 2–3 days. In October 2018, the patient began medication preparation for frozen embryo transfer according to the long protocol. On the 19th day of her menstrual cycle, gonadotropin-releasing hormone (GnRH) agonist 3.75 mg was administered intramuscularly. Sixteen days after the injection, the patient received estradiol valerate 2.0 mg, two tablets daily; folate 800μg, one tablet daily; and from the 16th day of the menstrual cycle, progesterone 200 mg, one tablet three times daily. According to ultrasound monitoring of the patient’s endometrium during the cycle, the thickness of the endometrium did not exceed 0.4 cm (Fig. 1). Therefore, the embryo transfer was canceled.

In November 2018, a diagnostic hysteroscopy, dissection of intrauterine synechiae, and endometrial biopsy were performed. According to a histopathological study, the endometrium was of the atrophic type; chronic endometritis was not confirmed immunohistochemically. For 4 months, the patient took various doses of estradiol valerate according to the different protocols, but her endometrial thickness did not change.

Given the situation, the use of a biotechnological product from the patient’s own endometrial cells was proposed. The patient agreed to the proposed treatment plan and signed the informed consent and all the necessary permits according to the Declaration of Helsinki and current Ukrainian legislation.

Before the endometrial biopsy, the patient was reexamined for human immunodeficiency virus, syphilis, hepatitis B virus, and hepatis C virus also underwent oncocytological examination of her cervix, urogenital smear microscopy, general blood and urine tests, liver tests, and coagulography to confirm the absence of infections and inflammatory processes. *Chlamydia trachomatis*, herpes simplex virus, cytomegalovirus, *Toxoplasma gondii*, and Epstein-Barr virus were tested by polymerase chain reaction and enzyme-linked immunosorbent assay. All the test results were normal. On the fourth day of the patient’s menstrual cycle, a pipelle biopsy (size 5 mm) was carried out from the inner mucosal layer of the uterus with subsequent transfer of endometrial material to a biotechnology laboratory.

**Fig. 2:**
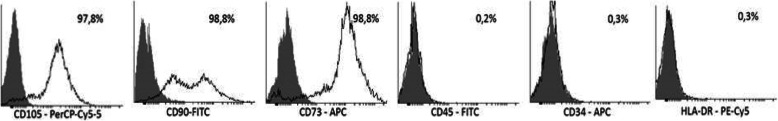
Immunophenotype profile of endometrial mesenchymal stem cells

Endometrial MSCs were isolated by enzymatic digestion and cultured under low-oxygen conditions (5% CO_2_ and 5% O_2_) and absolute humidity. The therapeutic dose of 20 million cells was expanded in 3 weeks. Before use, the cells were tested for the absence of infections (hepatitis B and C viruses, herpes simplex virus 1/2, cytomegalovirus, *Mycoplasma genitalium*, *Mycoplasma hominis*, and *Treponema pallidum*). The MSC identity was confirmed by fluorescence-activated cell sorting immunophenotype: CD90^+^CD105^+^CD73^+^CD34^−^CD45^−^HLA-DR^−^ (Fig. 2). The cells were capable of trilineage differentiation (adipogenic, osteogenic, and chondrogenic). The cells were frozen in liquid nitrogen before use.

**Fig. 3:**
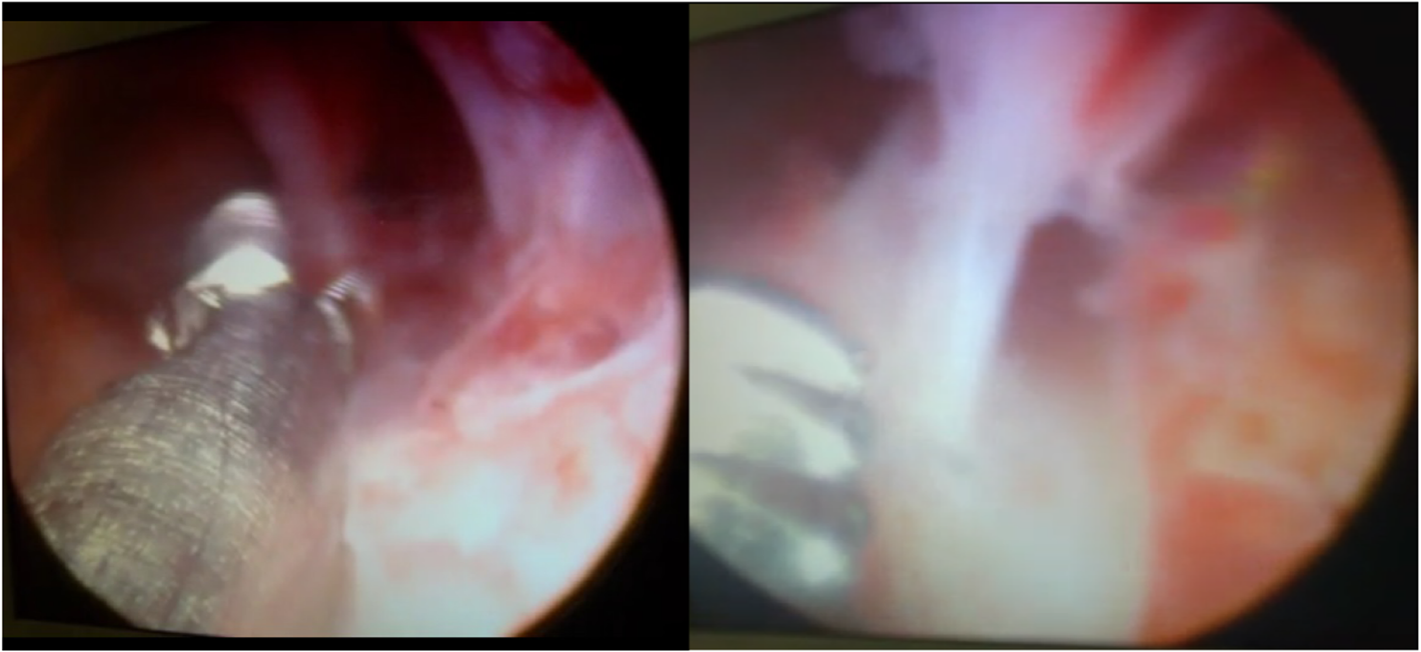
Hysteroscopy of intrauterine adhesions in the uterine cavity

On the 20th day of the patient’s menstrual cycle, she was administered GnRH agonist 3.75 mg intramuscularly. Sixteen days after the injection (on the fourth day of her next menstrual cycle), ultrasound monitoring of the endometrium and hysteroscopy were performed. According to ultrasound monitoring, her endometrial thickness was 0.2 cm, single-layered, and its structure was not visualized in the middle third of the uterine cavity. Diagnostic hysteroscopy, removal of intrauterine adhesions in the middle third of the uterine cavity (Fig. 3), and submucosal administration of cultured endometrial MSCs at a dose of 20 million cells were performed.

**Fig. 4:**
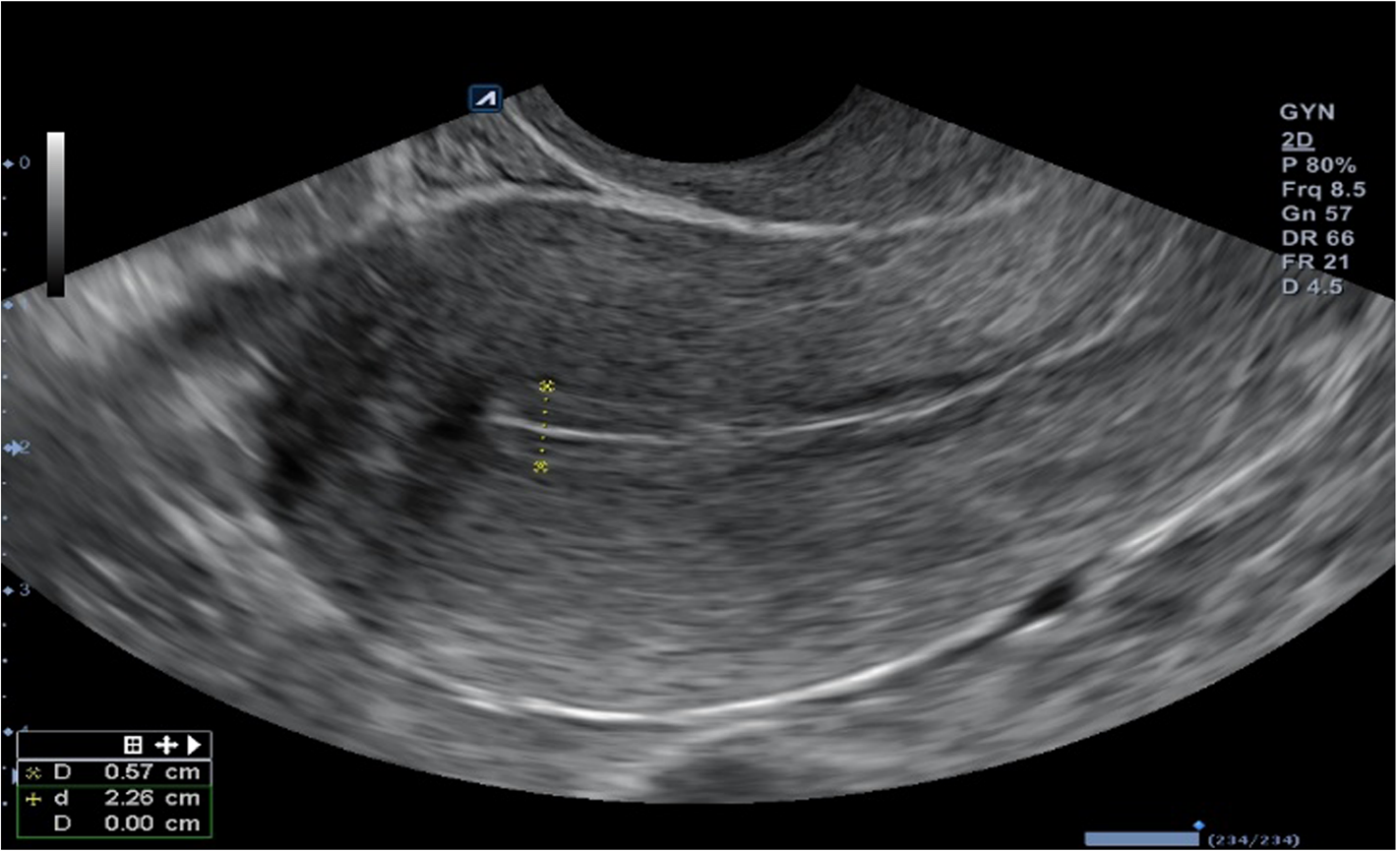
Ultrasound examination showing endometrial thickness in preparation for embryo transfer 13 days after endometrial mesenchymal stem cell transplantation

**Fig. 5:**
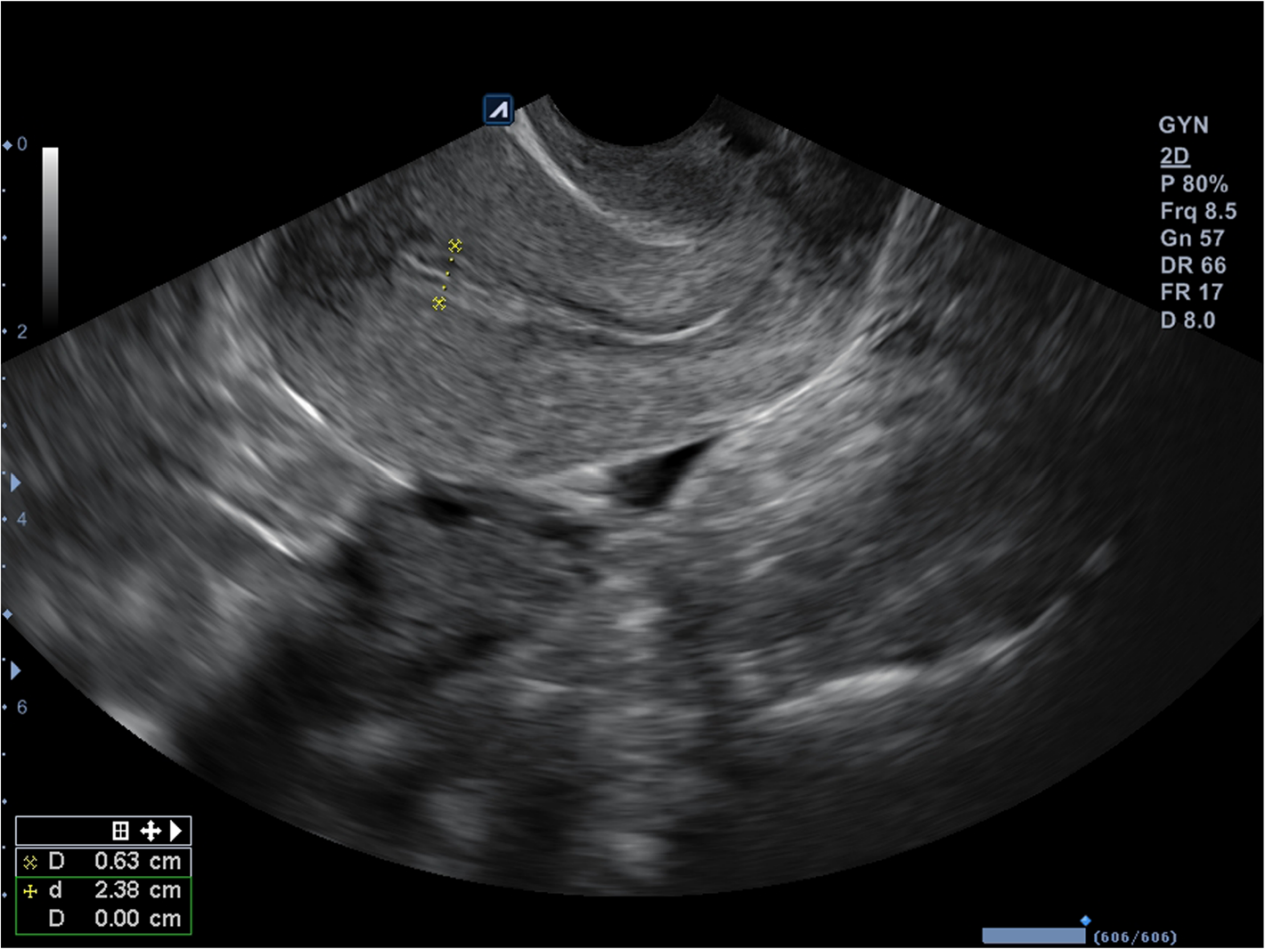
Ultrasound examination showing endometrial thickness on the day of embryo transfer

On the day of hysteroscopy, the patient began to take estradiol valerate 2.0 mg, at two tablets daily by mouth. After 3 days, a gel of estradiol hemihydrate 1.0 mg was added, one sachet daily transdermally. Thirteen days after hysteroscopy, ultrasound monitoring of the endometrium was performed, and progesterone 200 mg was prescribed, one tablet three times daily by mouth. On the sixth day of the progesterone application, the thickness of the patient’s endometrium was 0.63 cm after ultrasound monitoring (Figs. 4 and 5). At the patient’s insistence, two embryos were transferred that day.

**Fig. 6:**
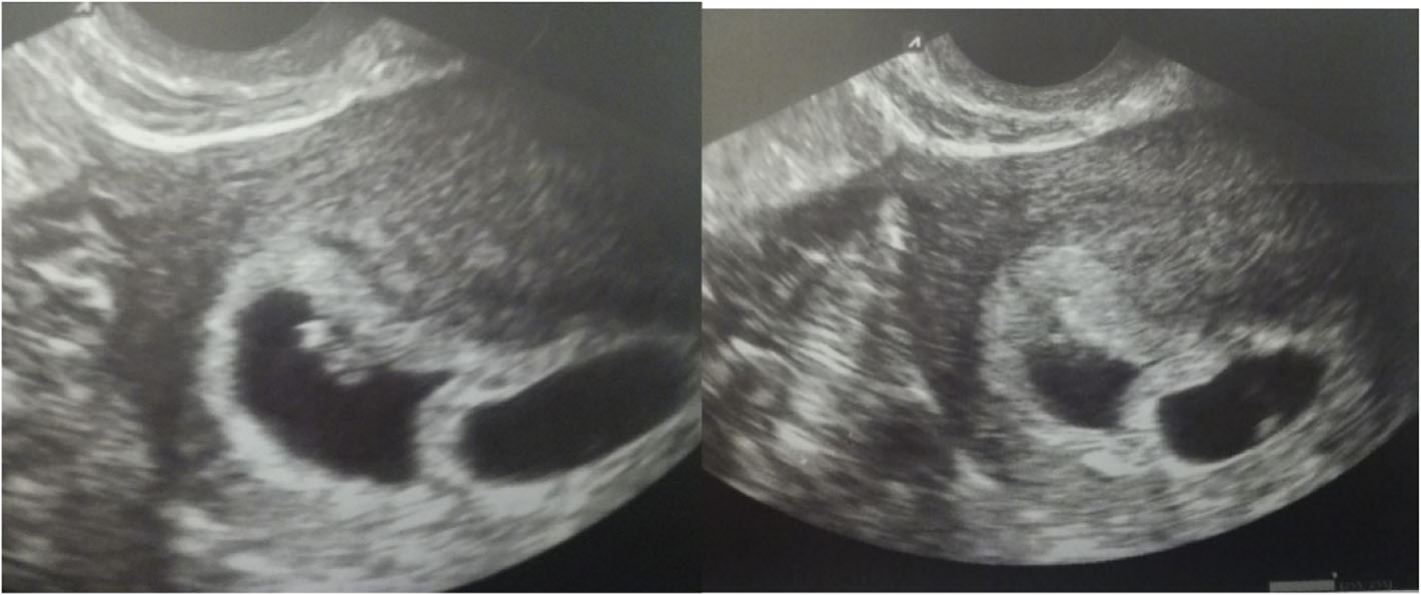
Ultrasound examination showing pregnancy confirmation

Two weeks after embryo transfer, the patient’s human chorionic gonadotropin level was 4795 IU/L. Four weeks after embryo transfer, ultrasound confirmed a dichorionic twin pregnancy (Fig. 6).

From 24 weeks of pregnancy, the patient was hospitalized at a perinatal center due to the threat of premature birth and intrauterine growth retardation of the second fetus. At 31 weeks of gestation, a cesarean section was performed because of rupture of membranes (amniorrhexis). Two girls were born, weighing 1280 g and 675 g, respectively. All the necessary stages of care for prematurely born children were carried out. On day 3, the girl with intrauterine growth retardation died. After 1.5 months, the woman and her other newborn girl were discharged to home. On the basis of observations of the pediatrician at 4 months after birth, the baby developed according to her age.

## Discussion and conclusion

The endometrium is a complex and dynamic tissue with unique properties of complete and fast self-renewal that happens, on average, once per month during a woman’s reproductive period. A thick, well-structured, and receptive endometrium is a must for successful conception. For a variety of reasons, some women experience problems with endometrial renewal that results in a thin endometrium and/or Asherman syndrome. A thin endometrium is a typical problem of impaired regeneration and thus is an issue for stem cell therapy [1]. There are numerous recommendations for the treatment of endometrial thickness using the possibilities of hormone replacement therapy. The effectiveness of such approaches is not high. Therefore, the search for new treatments for thin endometrial syndrome is quite relevant today.

The use of MSCs in regenerative medicine has gained increasing popularity in recent years due to outstanding therapeutic potential of this cell type. They stimulate the organism’s regenerative capacity in exerting a paracrine effect, producing cytokines and growth factors and thus pushing the on-site stem/progenitor cells and recruiting stem cells from other locations; besides, they are able to integrate into the damaged area [2].

Autologous endometrial MSCs are easily accessible during standard diagnostic procedures, have high proliferative and self-renewal capacities, are absolutely compatible with the recipient, and thus have more potential for on-site integration. After transplant, the cells provide structural and paracrine support to the damaged endometrium. We have previously shown that endometrial MSCs in culture produce vascular endothelial growth factor, granulocyte-macrophage colony-stimulating factor, basic fibroblast growth factor, interferon-γ-inducible protein 10, monocyte chemoattractant protein 1, interleukin-1 receptor antagonist, interleukin-6, interleukin-8, and interferon-γ [8]. We suppose that such a cocktail of growth factors, cytokines, and hormones could increase endometrial receptivity even if the thickness of the tissue is not yet in the optimal range, as described in the present clinical case. Of course, further randomized controlled clinical trials are absolutely necessary to prove the efficacy of this method.

## Data Availability

Data sharing is not applicable to this article, because no datasets were generated or analyzed during the present study.
